# Factors associated with nurses’ self-efficacy in oral care at Oulu University Hospital, Finland

**DOI:** 10.2340/aos.v83.42220

**Published:** 2024-11-06

**Authors:** Roosa-Maria Kivilahti, Tiia Ahomäki-Hietala, Hannu Vähänikkilä, Taru Aro, Vuokko Anttonen, Marja-Liisa Laitala, Anna-Maija Syrjälä

**Affiliations:** a Research Unit of Population Health, Faculty of Medicine, University of Oulu, Finland; b Northern Finland Birth Cohorts, Arctic Biobank, Infrastructure for Population Studies, Faculty of Medicine, University of Oulu, Finland; c Medical Research Center and Oulu University Hospital, Oulu, Finland

**Keywords:** Self-efficacy, oral care, infection-sensitive patients

## Abstract

**Objectives:**

This study aimed to investigate factors associated with nurses’ self-efficacy in oral care among infection-sensitive patients in a university hospital.

**Material and methods:**

All the nurses working in five wards of internal medicine and one department of oncology at Oulu University Hospital, Finland (*n* = 114) were recruited. Data were collected with a questionnaire containing 10 self-efficacy items (scored 0 to 10) and nine knowledge items (five-point Likert scale) that were validated in an earlier pilot study. Factor analysis was performed for the self-efficacy scale and a mean score was calculated for the knowledge scale. A multivariate linear regression model was used to analyze the association between explanatory variables and self-efficacy factor scores.

**Results:**

Factor analysis revealed self-efficacy factors: *Practical skills, Self-confidence in taking care of patients’ oral hygiene*, and *Confidence in detecting oral problems* (factor scores varied between 4.9 and 8.8). A higher mean score for the knowledge scale was positively associated with the factor *Practical skills* (*B* = 0.5, *p* < 0.05). Longer working experience was associated with higher factor scores in *Self-confidence in taking care of patients` oral hygiene* and *Confidence in detecting oral problems*.

**Conclusions:**

Better oral health-related knowledge and longer working experience were positively associated with oral health-related self-efficacy.

## Introduction

The microflora of the oral cavity is diverse and abundant, estimated to comprise more than 700 different species. Oral microbiota is associated with dental caries, periodontal infection and systemic diseases such as cardiovascular disease, cancer and diabetes [1]. Bacteria that colonize teeth and other oral surfaces also comprise a reservoir of respiratory pathogens [2], and aspiration of these respiratory pathogens is an important factor in the development of pneumonia in hospitals [3, 4]. In fact, oral hygiene strategies have been found to reduce pneumonia in hospitals [5–7, 8], lower the risk of postoperative infections, and enhance recovery in cancer patients [9].

Despite the obvious importance of patients’ oral care in everyday nursing practices, previous studies in hospital wards have shown that oral care is often inadequate and not a part of routines [10–13]. It has been reported that nurses in hospitals are aware of the link between oral health and systemic health [14–16], but a more profound oral health-related knowledge has been found to be lacking [14–18]. Furthermore, it has also been found that there is a discrepancy between knowledge of oral health and practices in oral care among nurses [15].

In addition to knowledge about oral health and the skills to manage daily oral hygiene practices among patients [19], nurses also need self-efficacy to overcome potential challenges in taking care of the oral health of patients. In 1977, Bandura defined the concept of self-efficacy as part of his socio-cognitive theory. According to the theory, human behavior is determined by the interaction between his/her personal factors, behavior, and his/her environment. The idea of self-efficacy is that a person’s belief in himself/herself and his/her abilities affect their actions and performance. The stronger one’s self-efficacy, the higher goals a person sets for himself/herself and the greater the will to work for his/her goals. Weak self-efficacy makes goal setting uncertain and reduces one’s commitment to these goals [20].

Self-efficacy in oral care and factors related to it have been previously analyzed among nurses providing oral care to older people [21–24]. The studies have indicated that the age [24] and working experience [23, 24] of nurses are associated with their self-efficacy in promoting oral care in nursing homes. In a study by Gu et al. [23], nurses with shorter working experience had higher self-efficacy in promoting oral care. Measuring self-efficacy has been found to be a useful way to analyze the improvement of oral care in nursing homes [24].

This study was carried out in university hospital inpatient wards where specialized medical care is offered to infection-sensitive patients among whom severe illnesses can be exacerbated by oral infections, making oral hygiene care essential. All participating nurses in this study, regardless of their educational background, are responsible for providing oral care for these infection-sensitive patients, especially if the patients are unable to perform it themselves. In specialized healthcare wards, the patients’ ability to maintain oral hygiene is often deteriorated due to severe illness and its therapy (e.g. surgeries, chemotherapy treatments). To our knowledge, this is the first study about nurses’ oral health-related self-efficacy in university hospital inpatient wards caring for infection-sensitive patients. Subsequently, the aim of this study was to analyze nurses’ oral health-related self-efficacy in patients’ oral care and factors related to it.

## Materials and methods

This is a questionnaire-based cross-sectional study where the entire nursing staff (auxiliary nurses, practical nurses, and registered nurses) working across five wards of the Oulu University Hospital, Finland, were recruited to participate. The education of registered nurses in Finland takes 3.5 years, being equivalent to a bachelor’s degree. The education of a practical nurse lasts about 2–3 years in Finland and prepares them for practice-oriented nursing tasks. An auxiliary nurse has the narrowest scope of education. Auxiliary nurses are no longer being educated, but they are still working in the nursing field.

Participating nurses were from the inpatient wards of oncology and hematology, cardiology, endocrinology and nephrology, pulmonology and infectious diseases. In these wards nurses care for patients with diseases that can be aggravated by oral infections and the majority of the patients in these wards are at a high risk of infection. The data were collected in August 2020. In the introduction provided to nurses before the survey, the nursing staff were asked to complete the questionnaire. The nurses answered anonymously and the introduction did not include any motivation to improve oral health-related self-efficacy or knowledge. The questionnaires were collected in a closed box on the wards and the time given to respond was 2 weeks.

### Questionnaire

The questionnaire was originally developed for geriatric home care nurses and piloted in a previous study [25]. Some modifications were made to make it more suitable for hospital settings.

### Outcome variable

#### Oral health-related self-efficacy factors

The questionnaire included 10 items to assess the nurse’s self-efficacy in carrying out the daily oral care of patients. Nurses answered on an 11-point scale with a score of 0 for the answer ‘I am not at all confident’ and a score of 10 for the answer ‘I am completely confident’. The dimensions of self-efficacy were analyzed using factor analysis.

Two items were excluded from the factor analysis to improve Cronbach’s alpha. The item ‘I am confident that I will make an appointment with a dentist for the patient I’m treating, or I will advise him/her to make an appointment himself/herself if he/she has a problem with his/her mouth’ was omitted from the analysis due to the low response rate to this item. The item ‘I am confident that I can detect the dryness of a patient’s mouth’ loaded onto both Factors 1 and 2, with a slightly stronger loading onto Factor 2. Omitting this item from Factor 2 improved Cronbach`s alpha from 0.650 to 0.697, enhancing the reliability of the results and the interpretation of Factors 1 and 2. Factor analysis revealed 3 factors, with corresponding Cronbach’s alphas of 0.797, 0.697 and 0.674 (Table 1).

**Table 1:** Self-efficacy items categorized into factors based on factor analysis with corresponding Cronbach’s alphas.

Factors and items (Cronbach’s alpha)	Factor loadings		
	I	II	III
*Self confidence in taking care of patient’s oral hygiene (0.797)*			
I am confident that I brush a patient’s teeth, even when pressed for time	0.842	-	-
I am confident that I clean a patient’s mouth, even if co-operation with him or her is problematic	0.658	-	-
I am confident that if a patient is unable to take care of their oral hygiene themselves, I look at their mouth every day	0.587	-	-
*Practical skills (0.697)*			
I am confident that I know how to brush a patient’s teeth	-	0.552	-
I am confident that I know how to clean a patient’s removable dentures	-	0.929	-
*Confidence in detecting oral problems (0.674)*			
I am confident that I am able to notice cavities in a patient’s teeth	-	-	0.431
I am confident that I am able to notice inflammation of the mouth	-	-	0.751
I am confident that I am able to notice mucosal inflammation or ulceration related to a patient’s use of removable dentures	-	-	0.591

### Explanatory variables

#### Oral health-related knowledge scale

The questionnaire included nine items about knowledge of oral health and its association with general health (Table 2). We changed the item related to knowledge ‘gingivitis is caused by bacteria in the mouth’ to the item ‘some drugs can reduce salivation’ to make the questionnaire more suitable for hospital settings. Nurses answered the items using a five-point Likert scale (completely disagree-disagree-no idea-agree-completely agree). The items were scored on a scale from 1 to 5, with 1 point assigned for an incorrect answer and 5 points for a correct answer. A sum variable was formed from the scores of the items and its mean score was calculated. The mean score of the knowledge scale was used as an explanatory variable in the analysis. Cronbach´s alpha for the sum variable formed from the knowledge items was 0.636.

**Table 2:** Frequencies (*n*) and proportions (%) of responses to items on oral health-related knowledge (*n* = 114).

Statements	Completely agree *n* (%)	Agree *n* (%)	No idea *n* (%)	Disagree *n* (%)	Completely disagree *n* (%)
I have enough knowledge about oral health	14 (12.3)	50 (43.9)	10 (8.8)	37 (32.5)	3 (2.6)
Oral health affects general health	103 (90.4)	8 (7.0)	0	0	3 (2.6)
Bleeding from the gums when brushing is normal	3 (2.7)	4 (3.6)	5 (4.5)	28 (25.0)	72 (64.3)
Halitosis can be caused by bacteria in the mouth	33 (29.2)	53 (46.9)	10 (8.8)	15 (13.3)	2 (1.8)
Poor oral health increases risk of aspiration pneumonia	51 (44.7)	29 (25.4)	31 (27.2)	3 (2.6)	0
Some drugs reduce salivation	101 (88.6)	9 (7.9)	2 (1.8)	1 (0.9)	1 (0.9)
Frequent consumption of sugar increases tooth decay	101 (88.6)	11 (9.6)	1 (0.9)	0	1 (0.9)
Chronic oral inflammation can increase the risk of dementia	26 (23.0)	15 (13.3)	64 (56.6)	6 (5.3)	2 (1.8)
Tooth loss is part of normal aging	3 (2.7)	9 (8.0)	9 (8.0)	28 (24.8)	64 (56.6)

#### Background factors

Sociodemographic and occupational factors were also asked. For the analysis, the participants were categorized according to their age (<30 years/30–39 years/40–49 years/≥50 years), basic educational background (basic education of 9 years/upper secondary education/third level education), nursing education (auxiliary nurse/practical nurse/registered nurse), years since graduation (<5/5–9/10–14/>14) and working experience (<5 years/5–9 years/10–14 years/>14 years).

### Statistics

The study population was described using frequencies and distributions according to their background variables. The knowledge items were described as frequencies (*n*) and proportions (%).

Factor analysis was performed for items measuring self-efficacy. The Kaiser-Meyer-Olkin Measure of Sampling Adequacy and Bartlett’s test were used to analyze the suitability of the data for factor analysis. The KMO was 0.818 and Bartlett’s test gave p < 0.0001. Three self-efficacy factors were formed. As self-efficacy is a continuous characteristic and there is no definite limit to low or high self-efficacy, self-efficacy was used as a continuous variable.

The association between background variables (age, basic education, education level in nursing, years since graduation, working experience) and mean scores for oral health-related knowledge (as explanatory variables), and mean scores for self-efficacy factors 1, 2, and 3 (as outcome variables) were analyzed using pairwise comparisons with univariate linear regression analysis. Explanatory variables that showed a statistically significant association with outcome variables in the bivariate analysis were selected for multivariate adjusted analysis. There was a strong correlation between the variables ‘years since graduation’ and ‘working experience’ and we preferred to select working experience for the multivariate model. Furthermore, basic education and nursing education did not show a statistically significantly association with the outcome variables in the bivariate analysis, but nursing education was considered an important variable to adjust for in the multivariate analysis.

Subsequently, a multivariate adjusted regression model was employed to examine the association between the explanatory variables, including age, nursing education, working experience, and the mean score of oral health-related knowledge and the outcome variables (mean scores for self-efficacy factors 1, 2, and 3).

Categorical background variables were converted into dummy variables for a linear regression model. Statistical analyzes were carried out with SPSS version 25.0 (SPSS, Inc., Chicago, IL, USA).

### Ethical considerations

The survey was voluntary and the participants gave their consent for participation by completing the questionnaire. Permission from the medical superintendent of the Oulu University Hospital was obtained for the study, and according to Finnish legislation, a statement from the Ethical Board was not needed.

## Results

The total number of respondents was 114, most of them registered nurses (*n* = 88, 77,2%). The largest age groups were those under 30 years old (*n* = 41, 36,0%) and 30–39 years old (*n* = 30, 26,3%). The largest working experience group was comprised of nurses with less than 5 years of working experience (*n* = 41, 36,0%). The second largest group was comprised of nurses with more than 14 years of working experience (*n* = 34, 29,8%) (Table 3).

**Table 3:** Background description of the study participants (*n* = 114).

Characteristic	*n*	%
Age (years)		
< 30	41	36.0
30–39	30	26.3
40–49	21	18.4
≥ 50	22	19.3
Basic education		
9 years	3	2.6
Upper secondary school	19	16.7
Third level education	92	80.7
Education level in nursing		
Auxiliary nurse	7	6.1
Practical nurse	12	10.5
Registered nurse	88	77.2
Years since graduation		
< 5	46	40.4
5–9	26	22.8
10–14	11	9.6
> 14	30	26.3
Working experience (years)		
< 5	41	36.0
5–9	23	20.2
10–14	14	12.3
> 14	34	29.8

Factor analysis of the self-efficacy scale revealed 3 factors; Factor 1: *Self-confidence in taking care of patients’ oral hygiene*, Factor 2: *Practical skills* and Factor 3: *Confidence in detecting oral problems*. The mean self-efficacy factor scores varied between 4.9 and 8.8; of the three factors, the factor *Practical skills* had the highest mean sum score of 8.8 (SD 1.1, min 5/max 10), and the factor *Confidence in detecting oral problems* had the lowest mean sum score of 4.9 (SD 1.6, min 0/max 9.7). The mean sum score of the factor *Self-confidence in taking care of patients’ oral hygiene* was 6.2 (SD 2.1, min 0/max 10). The items of the factors, factor loadings and Cronbach’s alphas are presented in Table 1.

Unadjusted and adjusted associations between explanatory variables and outcome variables analyzed by linear regression models are shown in Table 4. The adjusted multivariate linear regression model indicated that those having a higher mean sum score for oral health-related knowledge scale had a statistically significantly higher sum score for the self-efficacy factor *Practical skills* (*B* = 0.05, 95% CI: 0.1 to 1.0 *p* < 0.05). Those having <5 years or 10–14 years working experience had a lower sum score for the factor *Self-confidence in taking care of patients’ oral hygiene* when compared to those with over 14 years of working experience (*B* = -1.6, 95% CI: -3.2 to -0.1, *p* < 0.05 and *B* = -1.9, 95% CI: -3.5 to -0.4, *p* < 0.05, correspondingly). Those with 5–9 years working experience had a lower sum score for the factor *Confidence in detecting oral problems* when compared to those with over 14 years of working experience (*B* = -1.2, 95% CI = -2.4 to -0.0, *p* < 0.05).

**Table 4:** Linear regression model analyzing the association between explanatory variables and mean score for factors.

	Factor 1		Factor 2		Factor 3	
	Unadjusted B, (95% CI)	Adjusted B, (95% CI)	Unadjusted B, (95% CI)	Adjusted B, (95% CI)	Unadjusted B, (95% CI)	Adjusted B, (95% CI)
Age						
<30	1.0	1.0	1.0	1.0	1.0	1.0
30–39	-0.3 (-1.3 to 0.7)	-0.4 (-1.7 to 0.8)	-0.2( -0.7 to 0.3)	-0.4 (-0.9 to 0.2)	-0.1 (-0.9 to 0.6)	-0.3 (-1.3 to 0.7)
40–49	0.7 (-0.4 to 1.8)	-0.2 (-1.9 to 1.4)	-0.3 (-0.9 to 0.3)	-0.5 (-1.3 to 0.2)	0.9 (0.1 to 1.8) *	0.0 (-1.3 to 1.3)
≥50	0.6 (-0.5 to 1.7)	-0.8 (-2.5 to 1.0)	0.5 (-0.1 to 0.1)	-0.6 (-1.4 to 0.2)	0.7 (-0.2 to 1.5)	-0.1 (-1.4 to 1.2)
Basic education						
Basic education of 9 years	0.7 (-1.7 to 3.1)	-	-0.4 (-1.8 to 0.9)	-	0.1 (-1.8 to 2.0)	-
Upper secondary education	0.6 (-0.4 to 1.7)	-	-0.0 (-0.6 to 0.5)	-	0.5 (-0.3 to 1.3)	-
Third level education	1.0	-	1.0	-	1.0	-
Education level in nursing						
Auxiliary nurse	1.4 (-0.2 to 3.1)	0.5 (-1.6 to 2.6)	-0.1 (-0.9 to 0.8)	-0.1 (-1.1 to 0.9)	0.3 (-1.0 to 1.6)	-0.6 (-2.3 to 1.0)
Practical nurse	0.2 (-1.1 to 1.5)	0.6 (-0.8 to 2.0)	0.3 (-0.3 to 1.0)	0.3 (-0.3 to 1.0)	0.2 (-0.8 to 1.2)	0.3 (-0.7 to 1.4)
Registered nurse	1.0	1.0	1.0	1.0	1.0	1.0
Years since graduation						
<5	-**1.4 (**-**2.3 to** -0.4) **	-	0.2 (0.3 to 0.7)	-	-**1.0 (**-**1.7 to** -0.2) **	-
5–9	-**1.4 (**-**2.5 to** -0.3)**	-	0.6 (-0.0 to 1.2)	-	-**1.3 (**-**2.1 to** -0.4) **	-
10–14	-**2.0 (**-3.4 to 0.6) **	-	0.1 (-0.7 to 0.9)	-	-1.0 (-2.1 to -0.1)	-
>14	1.0	-	1.0	-	1.0	-
Working experience						
<5	-**1.2 (**-**2.2 to** -0.3)**	-**1.6 (**-**3.2 to** -0.1)*	0.2 (-0.3 to 0.7)	-0.4 (-1.1 to 0.4)	-**1.1 (**-**1.8 to** -0.3)**	-1.2 (-2.4 to 0.0)
5–9	-**1.2 (**-**2.2 to** -0.3)*	-1.2 (-2.8 to 0.3)	0.4 (-0.1 to 1.0)	-0.1 (-0.6 to 0.9)	-**1.3 (-2.2 to** -0.5)**	-**1.2 (**-**2.4 to** -0.0)*
10–14	-**1.8 (**-**2.3 to** -0.1)**	-**1.9 (**-**3.5 to** -0.4)*	0.5 (-0.2 to 1.2)	0.3 (-0.4 to 1.0)	-**1.0 (**-**2.0 to** -0.1)*	-1.1 (-2.2 to 0.1)
>14	1.0	1.0	1.0	1.0	1.0	1.0
Knowledge mean score	0.1 (-0.8 to 1.0)	-0.1 (-1.0 to 0.9)	0.6 (0.2 to 1.1)**	0.5 (0.1 to 1.0) *	0.07 (-0.6 to 0.8)	-0.1 (-0.7 to 0.8)

footnote: Factor 1: Self-confidence in taking care of patients’ oral hygiene, Factor 2: Practical skills, Factor 3: Confidence in detecting oral problems: Results from bivariate and multivariate analyses.

* *p* < 0.05,

** *p* ≤ 0.01

## Discussion

Our main results showed that nurses in university hospital wards treating infection-sensitive patients had the highest self-efficacy in *Practical skills* and the lowest self-efficacy in *Confidence in detecting oral problems*. Better oral health-related knowledge was positively associated with better self-efficacy in *Practical skills* and longer working experience was positively associated with higher self-efficacy in *Self-confidence in taking care of patient’s oral hygiene* and in *Confidence in detecting oral problems*.

Self-efficacy is an individual’s belief of how he/she manages to cope with challenges related to certain tasks [20], and nurses need self-efficacy to manage challenging situations in daily oral practices among patients. Our factor analysis revealed three self-efficacy factors, *Self-confidence in taking care of patients` oral hygiene, Practical skills* and *Confidence in detecting oral problems*, which describe the essential aspects of self-efficacy in oral hygiene care among nurses treating patients with an increased risk of infection. Improvement in self-efficacy in *Confidence in detecting oral problems* is especially warranted as the nurses in our study had the lowest self-efficacy in that factor.

To improve oral health-related self-efficacy among nurses, the sources of self-efficacy have to be identified. According to Bandura, the sources of self-efficacy include personal accomplishment, modeling of others, and verbal persuasion [20]. Hands-on training could be provided by oral healthcare professionals to hospital nurses and thus provide a model and enable the personal accomplishment of the nurses during hands-on training. Nurses’ oral health-related self-efficacy may also be improved by persuading them to improve their daily oral care of patients. One new result we found was that increased oral health-related knowledge is positively associated with greater self-efficacy in *Practical skills* related to oral care. Currently, the curriculum for nurses in Finland seems to include only minimal oral health-related training [26, 27]. More oral health-related education in the nursing curriculum, along with on-the-job training, is needed. Altogether, improvements in nurses’ self-efficacy in providing oral care to infection-sensitive patients are warranted.

In our study all participating nurses (practical nurses, auxiliary nurses, registered nurses) with different educational backgrounds are responsible for patients’ oral care, and there is no specific task allocation for patients’ oral care among the nurses. One interesting finding is that the equal responsibility in oral care is evident in the results in that the education level in nursing was not associated with the sum scores for three oral health-related self-efficacy factors. On the other hand, working experience was associated with *Self-confidence in taking care of patient’s oral hygiene* and *Confidence in detecting oral problems*. These findings suggest that extensive clinical years of work experience, but not nursing education, is strongly associated with better self-efficacy in providing oral care and identifying oral health issues.

Our results are partly contrary to a previous study where shorter working experience was found to be associated with higher self-efficacy in providing oral care among nurses whereas age was not associated with self-efficacy in that study [23]. The differing results between the previous study [23] and our study may be due to differences in measures of self-efficacy, institutional settings and differences in nursing education.

The primary reliability and validity of the scale for nurses’ oral health-related self-efficacy have been previously described in a pilot study but factor analysis was not performed in that study due to the small number of participants [25]. The strength of this study is that the factor analysis of the self-efficacy scale revealed the factors *Self-confidence in taking care of patients’ oral hygiene, Practical skills*, and *Confidence in detecting oral problems*, with sufficient factor loadings and Cronbach’s alphas for the factors showing acceptable internal consistency of the self-efficacy scale. Thus, the factor analysis supports the utility of the scale for analyzing self-efficacy among nurses in other studies as well.

A total of 114 nurses in all five wards participated in the survey. One limitation of the study is that the exact response rate could not be assessed because of the varying number of the staff (temporary and part-time staff, deputies for those on leave, working at the same time on different wards etc.) working in the wards. Furthermore, since answering the survey was entirely voluntary, it is possible that the participating nurses find oral health more important than layouts. Thus, social desirability has to be taken into account in the interpretation of the answers and may have skewed the results in a more positive direction.

## Conclusions

Our results showed that among nurses working in university hospital wards treating infection-sensitive patients, oral health-related knowledge was positively associated with self-efficacy in *Practical skills*. In addition, longer working experience was associated with better self-efficacy in *Self-confidence in taking care of patient’s oral hygiene* and *Confidence in detecting oral problems*.

## Data Availability

The data supporting the findings of this study are available from the corresponding author [R-MK] upon reasonable request.
